# Miscellaneous Notices

**Published:** 1848-10-01

**Authors:** 


### Miscellaneous Notices.

*Outlines of Lectures on the Nature, Cause, and Treatment of Insanity.* By Sir A. Morison, M.D. Edited by his Son, Thos. C. Morison, Esq. London: Highley. 1848.

We had intended fully analysing this work in our present number, but owing to a pressure of matter, we have been obliged reluctantly to defer its consideration for some future occasion. We cannot, however, permit the *Journal* to go to press without directing the notice of the profession to these Lectures. They will find the volume full of valuable information. The notes of the son will be read with great interest. In the next number of the *Journal of Psychological Medicine,* we propose doing ourselves the pleasure of reviewing in detail this treatise.

*Report of the Recent Progress of Psychological Medicine.* By C. Lockhart Robertson, M.D., Medical Staff, attached to the Royal Military Lunatic Asylum of Yarmouth, &c.

We have to acknowledge the receipt of this reprint from Dr. Ranking's half-yearly Abstract of Medical Sciences, vol. vii., 1848. The author is a gentleman of considerable talent, and bids fair to take a high position in his profession. He has paid much attention to the subject of insanity, and Dr. Ranking has shown much judgment in selecting Dr. Robertson to write the report of the progress of psychological medicine. He has performed his duty admirably. The article in question contains a faithful analysis of the works which have appeared in connexion with this subject, since the publication of the last volume of Dr. Ranking's annual abstract.

*Twenty-eighth Annual Report of the Dundee Royal Asylum for* 1848.

Since the publication of the last Report, fifty-two patients have been admitted into this asylum. In most of these cases the disease had not been of more than twelve months' duration. The deaths in the asylum have exceeded the general average. The report, on the whole, is extremely satisfactory. The tabular documents at the conclusion appear to be drawn up with great care.

*The Report of the Medical Officers of the Lunatic Asylum for the County of Lancaster.* 1848.

At the date of the last report, there were in this asylum 681 patients. During the past year, the admissions have amounted to 259 ; 116 have been discharged ; 59 have died ; making the present number of inmates 765. In comparison with former years, the number of admissions have been greater, and the mortality less. Since the last report, two additional wards on the male side have been opened for the reception of patients. On the subject of the schools established in this excellent asylum, the report says :—

"It is now more than six years since the first attempt was made to bring the influence of tuition into operation as a means of improving the inmates. In the first instance, we endeavoured to accomplish this object through the medium of a few of the better-informed and capable patients. It was thought that, when carried out in this way, the schools would serve a good purpose, not only in giving instruction to the ignorant, but also in providing a suitable occupation for the better educated and intelligent. For some time, one of the day rooms on each side of the building was appropriated to the purposes of a school-room, and the patients were conducted thither each morning. Considerable inconvenience arose from this arrangement, in consequence of the necessity of bringing patients through the different wards to school; and, moreover, the desirable influences of education could only be brought into operation on a very small portion of the inmates. After a while, it was determined to try how far evening schools could be made to serve the purpose in view. The experiment was first conducted in the winter months, among that class who work in the land, and who live together in a separate building. Some little difficulty was experienced, in making the attempt, to induce nearly all the inmates of a ward to enter upon school exercises. By a little perseverance and determination, however, this was at length surmounted, and the system was forthwith brought into operation in every ward throughout the establishment.

"It is the custom at Lancaster to keep the patients employed, as much as possible, up to four o'clock in the afternoon. At this hour, all work in the wards is put away, and the patients are encouraged to enter on different recreations and amusements. But, on those evenings appropriated to tuition, the patients are formed into different classes, and the instruction is conducted by the attendants and nurses of each ward, aided by a few more able patients, who act as monitors. During the past winter, we have derived much valuable assistance from the kind co-operation of the chaplain, Mr. Danby, who has materially contributed in forwarding this important work.

"In addition to the evening schools, the daily training of the idiotic forms a part of each morning's employment in one ward on each side of the house.'—p. 6.

The following is a summary respecting the ages of those patients in the asylum congenitally affected :—

| | No. under 25 years. | Average age of the whole. |
| --- | --- | --- |
| Females 106 | 26 | 39 |
| Males 107 | 35 | 34 |

"The result of this inquiry proves, as might naturally have been anticipated, that a great many of those born mentally imperfect, amongst the lower classes, are not brought into the returns until they have reached that period of life when, with an ordinary amount of faculty, they would have been able to maintain themselves. Whatever may be the circumstances which lead to this—whether it be from an unwillingness to report, on the part of the parents, from an undefined dread that their helpless children will be removed to the workhouse—or, whether it arise from a refusal on the part of relieving officers to render pecuniary aid on behalf of those so juvenile—the fact is certain, that the large body of idiotic paupers do not attract notice until they have passed the period of youth. They are brought forward and cast on the public, as a permanent charge, at a time of life when little hope is left of remedying their defects, and rendering them capable of contributing to their own support.

"If to the returns already made of those idiots who receive parochial relief, we were able to add the number of very young idiotic pauper children for whom assistance has not yet been sought, and if, also, we were able to ascertain the actual number belonging to that class of the poor placed just above pauperism, we should have a more just idea of the extent to which such mental imperfections prevail among the lower grades of society. There is no doubt that it would be necessary to make a very large addition to the figures 503, which, according to the existing returns, represent the numerical amount of the idiotic poor in Lancashire.

"In the State of Massachusetts, a thorough inquiry has lately been made on this subject, and it appears that out of 543 idiots visited by the commission, only 106 are supported at the public expense. Although the condition of the poor, in this county, and in Massachusetts, may be widely different, yet this fact may serve to give some idea how far the number of individuals now receiving parochial relief in Lancashire, on account of congenital mental imperfections, falls short of those for whom some further care and provision appear desirable."—p. 8.

It is lamentable to think that *juvenile* idiotic paupers are not brought under the cognizance of the Poor Law authorities, until the period of youth is past. Owing to this circumstance, we have reason to believe that idiocy prevails to a greater extent in this country than would at first sight be imagined. It is justly observed in the report that,—

"It is incumbent on those who are in any way responsible for these helpless beings, to endeavour to devise and establish the necessary means of rescuing them from a life of degradation, by training them up in orderly habits, and applying their small share of faculty to appropriate occupation."—p. 9.

The following extract in relation to the subject of idiocy will be read with interest:—

"There are some districts in the north of Lancashire, where idiocy is so prevalent as to give rise to the opinion that local causes contribute to its origin. In one particular district, comprising a population of 2250, it has been ascertained that there are no less than eleven idiots, the children of parents belonging to the agricultural and labouring classes, two only of whom are returned as paupers. Some investigation has been made in these villages, with a view of ascertaining whether any information could be derived which might throw light on the causes producing so striking a prevalence of congenital imperfections of mind. The parents are generally intelligent and industrious, and although living in a remote and sea-bound part of the country, where intermarriage might be supposed to be frequent, yet it did not appear that this cause had contributed to the degeneracy of offspring. In most instances, one child only in a family was found affected, the others appeared healthy, lively children, evidently capable of, and disposed to bestow, an affectionate care over their weaker brother. At the same time that such solitary examples appear to have arisen in different houses, yet it is singular that all the children in *one* family, amounting to no less than nine, were affected from birth. Both parents are intelligent, labouring people, they are not related by consanguinity, nor are they aware of any hereditary tendency to insanity in either family, the sole trace being that of slight eccentricities in one of the ancestors. Only one child out of the nine is now living, the others having died at various ages between infancy and manhood, but all surviving long enough to leave no doubt that each was born mentally deficient. Had all these children survived, the proportion of the idiotic in this particular district would have been considerably increased.

"Before leaving this subject, it may be as well to notice, that a still greater amount of idiocy is found to prevail among the poor who live in some of the secluded dales formed by the range of hills separating Lancashire from Yorkshire. In one particular locality, the proportion is so high as to reach one in a hundred of the inhabitants. And it may be remarked, that here intermarriage has evidently formed one of the circumstances which have given rise to this degeneracy. But not to dwell further on the causes of imperfect cerebral development, it is satisfactory to think that the means of remedying such defects are now practised with success; and therefore, it has been thought desirable rather to direct attention to the alleviation of the evil, than to devote much time to the investigation of its causes."—pp. 9, 10.

It would appear, according to a return of the number of idiotic and insane paupers in the different townships of the county of Lancaster, made by the Poor Law medical officers in 1847, that there were—

| | |
|---|---|
| Idiots | 198 |
| Imbeciles | 305 |
| Insane | 185 |
| Total | 688 |

*The Ethnological Journal.* Edited by LUKE BURKE, Esq. Nos. 1, 2, 3, and 4. London: (monthly.) Red Lion Court, Fleet-street.

WE give this new periodical a most friendly greeting. The subject which Mr. Burke has proposed to elucidate, is one of much interest and importance. The articles which have appeared in the magazine are happily selected, and are written with ability. The journal is deserving of success. We are pleased to hear that its circulation is increasing both at home and abroad. We hope ere long to consider somewhat in detail the topics discussed in the numbers before us. For the present we must be satisfied with giving it a hearty welcome.

*The Morning Side Mirror.*

WE have, through the courtesy of our Scotch friends, received several copies of this interesting little periodical, written and printed in the Morning Side Asylum for Lunatics. The profits of this publication are devoted to the reading-room of the asylum. The articles are well written, showing talent, spirit, and wit. From a number before us we select the following extract of interest:—

"In the heart of the Alps, and at some four thousand feet above the surface of the sea—surrounded by all the grandeur of Swiss scenery, and beyond the noxious influences which render insalubrious many of the most beautiful spots of that land of heroes and poets, like some out-post of that civilization and philanthropy, the essence of which it so fully represents—lies the Hospital of Abendberg, the first, and, we believe, as yet, the only establishment founded with the avowed purpose of curing Idiocy. This malady, which is very prevalent in Switzerland, is associated with, if not caused by, various diseases of a purely physical nature, such as scrofula, rickets, and bronchocele; takes the name of *Cretinism;* and, like its concomitants, is transmitted from parents to their offspring, assuming forms the most repugnant, and exhibiting humanity in its most degraded condition.

"From motives purely scientific, as much as from the humane and truly Christian tendency of such an institution, we should not hesitate to undertake a difficult journey, and would think lightly of many a sacrifice which should enable us to visit an establishment having so many claims to notoriety, and possessing so much intrinsic merit. Without having been called upon to perform any such act of self-denial, we have been, through the kindness of our Resident Physician, favoured, not with a sight and inspection of Abendberg, but with that which we consider of greater interest still, a few hours' conversation with its celebrated projecter and founder, Dr. Guggenbühl. Fain would we treat our readers to a glance into the benevolent and ingenious mind of the gifted individual by whose earnest and sustained labours science and humanity have gained so much; but as the task would require further opportunities, besides a proportionate amount of talent, to which we can lay no claim, we shall content ourselves with a few observations gathered from his conversation; being moreover, assisted by a pamphlet on the subject, from which we hope to gather some information not uninteresting to our readers.

"As affording the best idea of the truly Christian spirit which animates our kind and benevolent doctor, we cannot do better than translate the following passage, occurring in one of his letters, published in 1846 :—

"'To improve the deplorable condition of these unhappy beings, and, with the assistance of God, to form and instruct them, until they become useful members of society, is nothing less than accomplishing an intellectual resurrection; it is in our epoch the greatest miracle of Christian charity, and one of the most noble missionary labours undertaken in the service of God. Let him not boast of his Christianity who does not feel himself disposed to do his best towards furthering this end: he is a man devoid of heart, and his soul is a prey to a species of Cretinism far more dangerous than that which attacks the physical and moral nature of these poor children.'

"To prove the importance of his subject, Dr. G. has collected, from various sources, information which goes to show that even in Austria, Cretinism has become so prevalent along the margins of the Danube, large and populous parishes are to be found, where, at the annual recruitment, not a single individual can be had capable of bearing arms. In many parts of Switzerland and Germany there is scarcely a family that is not burthened with at least one of these unhappy and helpless beings; while many families are to be met with composed altogether of cretins and half-cretins.

"It is evident from the above, that one hospital in Switzerland, containing only about forty children, is far from being sufficient to relieve the amount of disease which such statements indicate; nevertheless, Dr. Guggenbühl's Institution has, since its establishment, greatly ameliorated the sanitary condition of the country. Many of the children, who are often taken in at the early age of six months, are soon cured, or so far improved as to enable their parents to carry on the same system of education, for which purpose they are supplied with ample directions from the doctor. None of them are kept beyond the age of seven years, it being found that, after this period, little can be done for such as are proof against all previous treatment.

"Besides the therapeutic and hygienic treatment, which partakes more or less of the plan followed in all cases of analogous disease, particular attention is paid to the intellectual and moral. This consists in 'giving the child a consciousness of the principles which form the foundation of its faculties, and in otherwise favouring the development of the intelligence. We begin by leading him into comparing, and the perception of analogies, and differences between various objects; and this is done by proceeding according to degrees of size, colour, form, substance; and next we resume these judgments in detail, and proceed to the formation of a general idea; and, thereafter, to certain conclusions drawn from these general ideas. When, by previous efforts, we succeed in awakening in the child the sentiment, that the existence of visible and limited matter reposes alone on the idea of the eternal and infinite—that the good and the just alone are agreeable to the Deity, while evil and injustice are displeasing to him,—then we see *reason* unveil and manifest itself.'

" 'This sentiment,' adds Dr. Guggenbühl, 'obtains great predominance with a great number of children at the moment when the mind awakens from its lethargy; and we have for a long time observed that their conception of the existence of God precedes the comprehension of such objects as fall within the sphere of their senses, such as a table and the like. It is the same with the sentiment they experience, that God manifests his presence in the phenomena of nature, so varied and so magnificently laid before their eyes in the neighbourhood of Abendberg. It is necessary to have witnessed the amazement, the joy, and the admiration of these children on regarding the sun rising and setting, the rainbow, or while listening to the reverberation of the thunder, in order to comprehend the truth of Diesterweg's saying, that "many an adult might be ashamed of his indifference and moral torpor in the presence of the phenomena of nature, when he finds himself beside the innocent child whose attention is thereby charmed, and whose joy and admiration know no limits." '

" That the labours of Dr. Guggenbühl have not failed in reaping the harvest which every well-intended and sustained effort must needs produce, may be gathered from the following extracts, translated from his letters, published in Zurich, and embodied in the *Archives des Sciences Physiques et Naturelles.*

" 'Many of our beloved pupils, who returned to their homes two years ago, have suffered no relapse, and their faculties have become sufficiently developed to enable them to follow the public schools with success.' Farther confirmation of the above may be found in the following, written in 1847, and a year subsequent to our first extract: 'Since the commencement of our labours, we may consider this summer as the most interesting period. This we owe both to the activity and devotion of our assistants, and to the creation of a species of institute for the mothers of children affected with cretinism, who are desirous of becoming acquainted with the details of its treatment.' And again—'Since the publication of my last report (1846), a good number of pupils have left the establishment; the majority of them had mastered their elementary instruction, and were, physically speaking, sufficiently developed to enable them to embrace a profession. The worst cases have been improved; and there has been no death. Our experience continues to prove that the caprices of these children soon give place to amiable sentiments, affection, and gratitude. There are some at present who write very pretty letters to their parents, and have a very remarkable knowledge of geography, natural history, &c. &c. I am more and more convinced that the spectacle of the phenomena of nature furnish the most efficient means to call into activity their torpid faculties, since these phenomena are of daily recurrence in our beautiful locality, and supply the mind at every moment with a new stimulus. At present I am engaged in experiments relative to the action of several vegetables calculated to combat this disease, and which grow in abundance in our vicinity.' . . . .

" We might add much on this comparatively new subject, both from the work before us, and the conversation of Dr. Guggenbühl, to which we have listened with the greatest interest; but it would far surpass the limits of this notice, and might possibly tire the patience of at least some of our readers. We have only to add, in reference to his visit to this establishment, where he spent the best part of the day, that he expressed himself highly pleased with its internal arrangements, and present condition; and having no reason to complain of these ourselves, we do not say Amen, and bow to his better judgment in such matters. After tea he repaired with Dr. Skae and ourselves to the ball-room, it being one of our *assembly nights;* and here, again, we were delighted to find that the strict good order and harmony observed, were to him so many additional sources of delight; and we were no less pleased with his remark, that although he had visited the most important establishments of the kind in Europe, he had nowhere found more order and regularity, or a more complete application of the enlightened methods of treating a malady in which the body of the material organ, not the mind, the immaterial essence, is at fault. He holds the same opinion in reference to cretinism, and hence his self-devotion, his almost missionary, or *more* than missionary zeal, and religious fervour in his praiseworthy efforts to awaken the soul from a lethargic indolence and insensibility to its vital interests, for which, in its abstract existence, it has no absolute relation; having never been intended by our all-beneficent Creator to drag on that less than vegetative life which our ignorance, or worse, have entailed upon so many of the children of men."

---

